# Nuclear RNA transcript levels modulate nucleocytoplasmic distribution of ALS/FTD-associated protein FUS

**DOI:** 10.1038/s41598-022-12098-4

**Published:** 2022-05-17

**Authors:** Yueh-Lin Tsai, Yu Chun Mu, James L. Manley

**Affiliations:** Department of Biological Sciences, Columbia University, New York, NY 10027 USA

**Keywords:** RNA-binding proteins, Nucleotide-binding proteins

## Abstract

Fused in Sarcoma (FUS) is a nuclear RNA/DNA binding protein that mislocalizes to the cytoplasm in the neurodegenerative diseases ALS and FTD. Despite the existence of *FUS* pathogenic mutations that result in nuclear import defects, a subset of ALS/FTD patients display cytoplasmic accumulation of wild-type FUS, although the underlying mechanism is unclear. Here we confirm that transcriptional inhibition, specifically of RNA polymerase II (RNAP II), induces FUS cytoplasmic translocation, but we show that several other stresses do not. We found unexpectedly that the epitope specificity of different FUS antibodies significantly affects the apparent FUS nucleocytoplasmic ratio as determined by immunofluorescence, explaining inconsistent observations in previous studies. Significantly, depletion of the nuclear mRNA export factor NXF1 or RNA exosome cofactor MTR4 promotes FUS nuclear retention, even when transcription is repressed, while mislocalization was independent of the nuclear protein export factor CRM1 and import factor TNPO1. Finally, we report that levels of nascent RNAP II transcripts, including those known to bind FUS, are reduced in sporadic ALS iPS cells, linking possible aberrant transcriptional control and FUS cytoplasmic mislocalization. Our findings thus reveal that factors that influence accumulation of nuclear RNAP II transcripts modulate FUS nucleocytoplasmic homeostasis, and provide evidence that reduced RNAP II transcription can contribute to FUS mislocalization to the cytoplasm in ALS.

## Introduction

Fused in Sarcoma (FUS) is an RNA/DNA binding protein predominantly localized in the nucleus and which functions in various nuclear processes such as transcription, splicing and DNA repair^1–3^. FUS cytoplasmic mislocalization is a pathological feature in a subset of Amyotrophic Lateral Sclerosis (ALS) and Frontotemporal Dementia (FTD) patients^4,5^. Mislocalization can be caused by mutations in the nuclear localization signal (NLS) of FUS, which disrupt its interactions with a nuclear import receptor and lead to import defects^6^. However, cytoplasmic mislocalization of wild-type (WT) FUS can also be observed in ALS and FTD patients^5,7^, suggesting the existence of an imbalanced nucleocytoplasmic homeostasis in diseased cells.

External stress stimuli have been shown to modulate FUS nucleocytoplasmic transport. Since FUS cytoplasmic accumulation is observed in ~ 9% of FTD patients^8^, and has also been reported recently in sporadic ALS patients^5^, investigating stresses that can induce FUS nuclear export may bring insights into the mechanisms of its homeostasis. Hypertonic stress in certain cell types such as neurons and HeLa cells has been reported to elicit FUS cytoplasmic redistribution^9,10^. The cell type-dependent FUS redistribution in response to hypertonic stress appears to correlate with the distribution of the nuclear import receptor TNPO1^10^. Under normal circumstances, TNPO1 shows a diffuse nucleocytoplasmic distribution and transports FUS from the cytoplasm to nucleus via binding to the FUS NLS. Hypertonic stress treatment of neuronal cells results in TNPO1 cytoplasmic accumulation, while TNPO1, and FUS, distribution in astrocytes remain unchanged^10^. High levels of glutamate that mimic excitotoxicity also induce FUS cytoplasmic translocation in cultured neurons^11^. Excess glutamate triggers Ca^2+^ to enter cells and it was shown that elevated Ca^2+^ levels alone are sufficient to induce FUS cytoplasmic translocation, possibly through redistribution of nucleocytoplasmic shuttling factors CRM1 and RAN^11^.

A number of studies have examined the effects of various stresses on FUS subcellular localization, albeit with mixed results. For example, increased DNA damage and oxidative stress have been implicated in ALS and FTD^12–15^, but studies examining FUS nuclear export in response to these stresses are still inconclusive. It was reported that FUS is phosphorylated by DNA-PK at the N-terminus and translocates to the cytoplasm after DNA damage induced by staurosporine or calicheamicin γ1 in H4 neuroglioma cells^16^, whereas another study reported that FUS remains nuclear under the same stress conditions in the same cell type^17^. Oxidative stress induced using H_2_O_2_ was shown to cause FUS cytoplasmic translocation after prolonged treatment (~ 90 min) in HeLa cells, but FUS actually showed more complete nuclear localization at an early time point (~ 15 min) after H_2_O_2_ addition, likely due to PAR-dependent FUS recruitment to DNA damage sites^18^. Other studies using sodium arsenite to induce oxidative stress for 1–2 h did not detect FUS cytoplasmic translocation^9,10^. Reduced levels of transcription were initially suggested to lead to FUS cytoplasmic localization. Transcriptional inhibition using 5,6-dichloro-1-beta-ribo-furanosyl benzimidazole (DRB), alpha-amanitin or actinomycin-D (Act-D) was shown some time ago to elicit FUS cytoplasmic translocation in HeLa cells^19,20^. Since FUS is an RNA binding protein (RBP) that associates with pre-mRNAs^21^, reduced transcriptional activity may release FUS into the nucleoplasm and favor cytoplasmic translocation. However, more recent studies, in both HeLa cells and human neurons, reported that FUS distribution remained unchanged after Act-D treatment^10,22^. Altogether, FUS nuclear export appears likely to be elicited by certain stresses, although the limiting factors remain unclear.

Here we report studies providing new insights into the mechanism of FUS nucleocytoplasmic homeostasis. We first subjected cultured cells to a variety of different stresses, including transcriptional inhibition. We show that the global transcriptional inhibitor Act-D and the RNA polymerase II (RNAP II) elongation inhibitor flavopiridol (flavo) lead to FUS depletion from the nucleoplasm and cytoplasmic translocation. Unexpectedly, we observed different degrees of FUS cytoplasmic translocation following transcriptional inhibition depending on the FUS antibody used, findings that explain the inconsistent previous results of FUS nucleocytoplasmic localization after transcriptional inhibition. Supporting the idea that levels of nuclear RNAP II transcripts can influence FUS localization, we found that depleting either the mRNA export factor NXF1 or the nuclear exosome cofactor MTR4 prevents FUS cytoplasmic translocation following flavo treatment. In contrast, mislocalization was independent of both the nuclear protein export receptor CRM1 and the import factor TNPO1. Finally, we investigated the possibility that reduced global RNAP II transcription occurs in ALS patient cells, and found that reduced expression of nascent transcripts, especially transcripts known to be bound by FUS, occurs in sporadic ALS iPS cells compared to normal controls. Together, our results link reduced RNAP II transcriptional activity/nuclear transcript levels to increased FUS cytoplasmic accumulation, thereby suggesting a possible mechanistic basis for the cytoplasmic localization of WT FUS observed in a subset of ALS/FTD patients.

## Results

### Transcriptional inhibition and hyperosmolarity, but not other stresses, induces FUS cytoplasmic translocation

**Figure 1:**
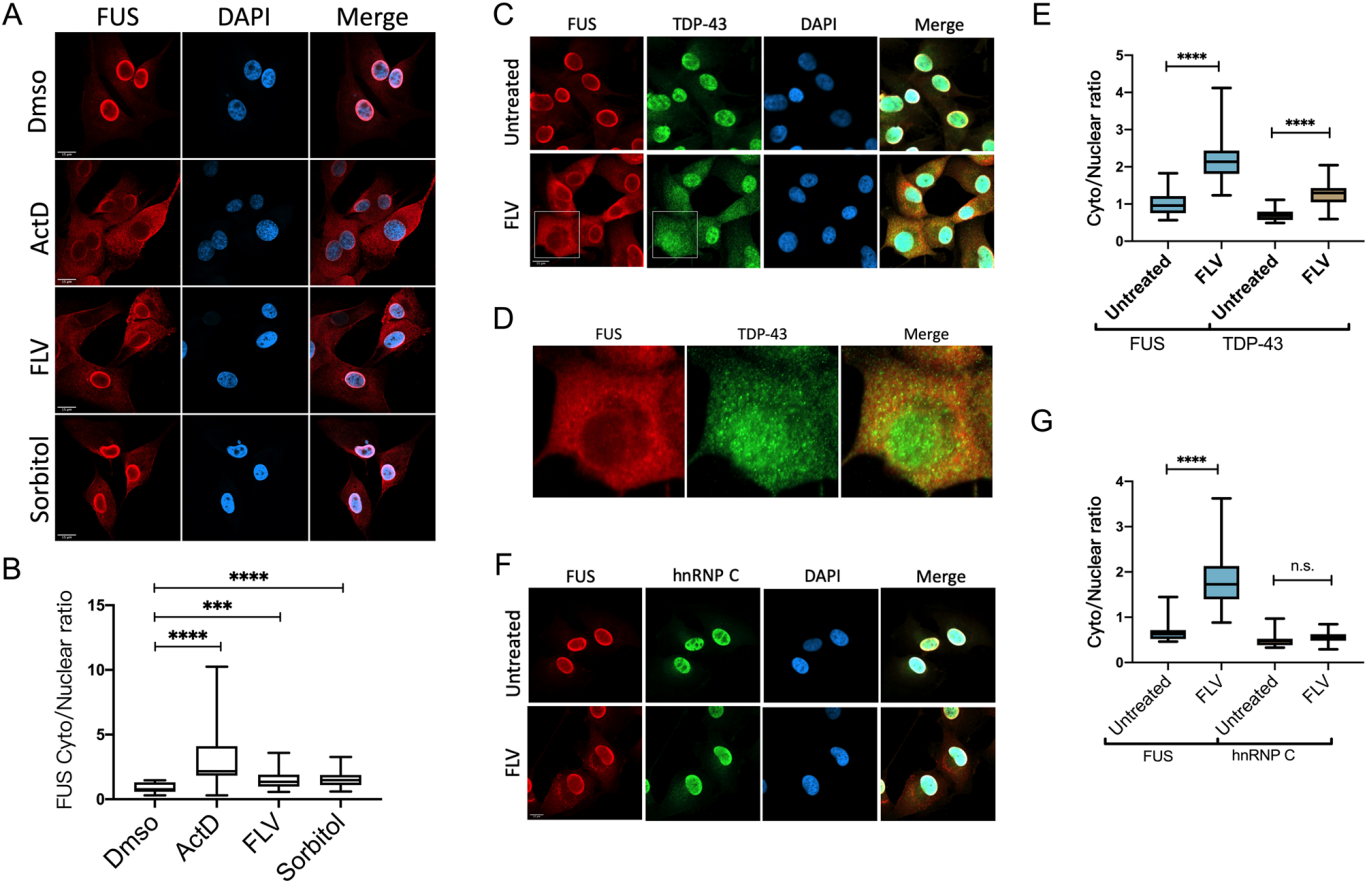
Transcriptional inhibition and hyperosmolarity induces FUS cytoplasmic translocation. (**A**) U87 cells were incubated with actinomycin D (ActD, 5 ug/ml), flavopiridol (FLV, 1 uM) or sorbitol (0.4 M) for 4 h, followed by paraformaldehyde fixation and FUS immunofluorescence (IF) using primary anti-FUS antibodies. Samples were washed, stained with secondary antibody, mounted and sealed for confocal imaging. (**B**) Cytoplasmic to nuclear FUS ratios were compared with the DMSO control. n = 24–33 cells were analyzed in each experimental condition. (***p = 0.0003, ****p < 0.0001) Mann–Whitney *U*-test. (**C**) U87 cells were incubated with 1 uM flavo for 4 h followed by FUS and TDP-43 IF. (**D**) Magnified images of FUS and TDP-43 co-staining. (**E**) Cytoplasmic to nuclear ratios were quantified for each protein in (**C**). n = 11–17 cells were analyzed in each experimental condition. (****p < 0.0001) Mann–Whitney *U*-test. (**F**) U87 cells were incubated with 1 uM flavo for 4 h followed with FUS and hnRNP C IF. (**G**) Cytoplasmic to nuclear ratios were quantified for each protein in (**F**). n = 15 ~ 18 cells were analyzed in each experimental condition. (****p < 0.0001) Mann–Whitney *U*-test.

As described above, it is not uncommon in ALS for WT FUS to localize to the cytoplasm. To investigate possible underlying mechanisms, we first examined whether different cellular stresses lead to FUS nuclear export. To this end, we examined FUS localization in human U87 glioblastoma cells by immunofluorescence (IF) following exposure to different stresses, specifically doxorubicin (DNA double strand breaks, DSBs), sodium arsenite (oxidative stress), sorbitol (hypertonic stress), serum deprivation, and flavo or Act-D (transcriptional inhibition). We observed striking FUS nuclear export following transcriptional inhibition using either inhibitor (Fig. 1A,B). Separation of cell extracts into nuclear and cytoplasmic fractions followed by western blot (WB) with anti-FUS antibodies confirmed the IF results (Supplemental Fig. S1). Hypertonic stress also induced FUS nuclear export (Fig. 1A,B), consistent with previous studies^9,10^. In contrast, DSB induction did not significantly alter FUS nucleocytoplasmic localization, while oxidative stress actually resulted in more complete FUS nuclear localization (Supplemental Fig. S2A, B).

With respect to oxidative stress, we observed prominent G3BP1-positive stress granules (SGs) following sodium arsenite treatment, suggesting that our protocol was sufficient to induce a robust oxidative stress response (Supplemental Fig. S2C). This is consistent with the PARP-dependent FUS recruitment to DNA damage sites observed at short times following exposure to H_2_O_2_^18^. We noticed that although sodium arsenite treatment induced FUS granule formation, the pattern was distinct from the G3BP1-positive SGs, i.e., FUS granules were not as prominent and prevalent (Supplemental Fig. S2B). To investigate whether the lack of FUS recruitment to SGs is cell type-dependent, we performed FUS and G3BP1 IF in primary human fibroblasts treated with sodium arsenite (Supplemental Fig. S2D). We observed both FUS and G3BP1 granule formation, but the overlap between FUS granules and G3BP1-positive SGs was partial. This observation is consistent with previous studies in neuronal cells^10,23^, suggesting that wild-type FUS is not a constitutive SG component.

As ER stress is also significantly increased in ALS and FTD^24,25^, we also examined whether FUS undergoes nuclear export when cells are incubated in low concentrations of serum (0, 0.1, 10% FBS) for 6 h. However, we did not observe a significant correlation between FUS nucleocytoplasmic distribution and serum concentration (Supplemental Fig. S2E).

We next compared FUS cytoplasmic translocation following flavo treatment with the behavior of two other proteins, the ALS/FTD-associated RBP TDP-43 and a non-shuttling RBP, hnRNP C. Consistent with a previous study^22^, IF revealed that TDP-43 underwent substantial cytoplasmic translocation upon transcriptional inhibition (Fig. 1C,E). Notably, FUS and TDP-43 did not co-localize after flavo treatment, indicating differential cytosolic localization of the two proteins (Fig. 1D). In contrast, hnRNP C did not show cytoplasmic translocation upon flavo treatment (Fig. 1F,G).

**Figure 2:**
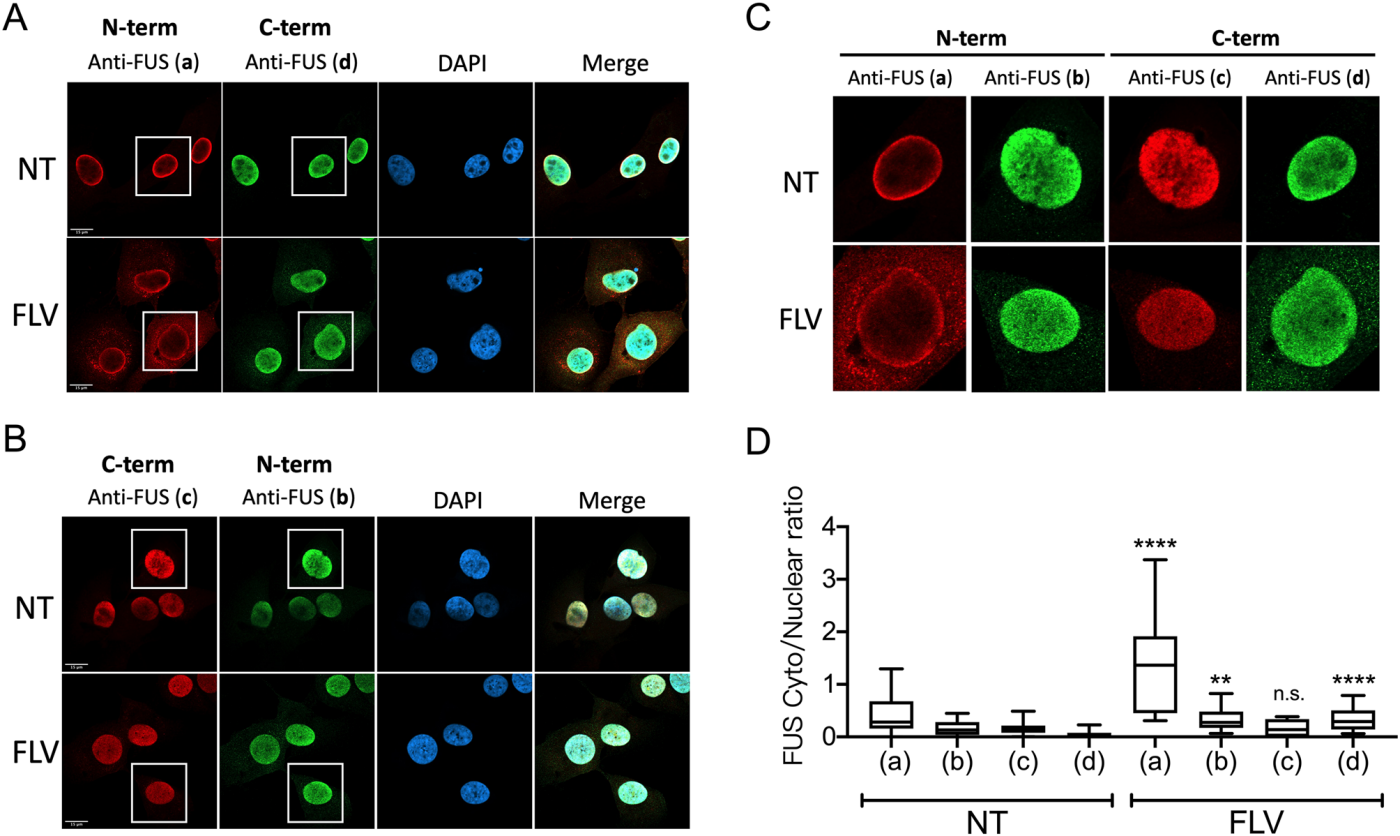
Transcriptional inhibition induces FUS cytoplasmic translocation demonstrated by staining with multiple antibodies. (**A**, **B**) U87 cells were treated with flavo (1 uM, 4 h) followed by fixation and FUS IF. Each of the two anti-FUS antibodies recognizing the N-terminus (**a**, **b**) were mixed with one of the two anti-FUS antibodies recognizing the C-terminus (**c**, **d**) as indicated for FUS IF. The primary anti-FUS antibodies were distinguished by fluorescence-conjugated secondary antibodies recognizing different species (red: mouse; green: rabbit). Final concentration of each primary antibody was 2 ug/ml. (**C**) Enlarged images of insets indicated in panel A and B. (**D**) Cytoplasmic to nuclear FUS ratios were compared between flavo-treated (FLV) and untreated control (NT) cells using anti-FUS N-terminus and C-terminus antibody fluorescent intensities. n = 13–26 cells were analyzed in each experimental condition. (**p = 0.0088, ****p < 0.0001) Mann–Whitney *U*-test. Scale bars = 15 µm.

Previous studies have reported different effects of transcriptional inhibitors on FUS nuclear egress. Zinszner et al. (1994; 1997) observed significant FUS cytoplasmic translocation after DRB or Act-D treatment, while Ederle et al.^22^ and Hock et al.^10^ detected only slight or no FUS cytoplasmic translocation with the same inhibitors^10,19,20,22^. One difference in these analyses was that Zinszner et al. used only FUS antibodies raised against an N-terminal epitope, but Ederle et al. and Hock et al. both included antibodies that recognize the FUS C-terminus. We therefore next investigated whether apparent FUS cytoplasmic translocation following transcriptional inhibition was dependent on the identity of the antibody used. To this end, we mixed either of two different FUS antibodies recognizing the N-terminus (designated a and b) with either of two antibodies recognizing different C-terminal epitopes (designated c and d) and performed IF in U87 cells that were either untreated or treated with flavo (Fig. 2A–C; antibodies were distinguished with different secondary antibodies). The measured cytoplasmic FUS percentages were adjusted for background staining of secondary antibodies (Supplemental Fig. S3A), while the specificities of all four antibodies were validated by depleting FUS with siRNA prior to IF, which in all cases strongly reduced signals (Supplemental Fig. S3B–E). While both N-terminal antibodies showed significant increases in cytoplasmic FUS following flavo treatment (twofold or greater; Fig. 2C,D), one C-terminal antibody failed to detect increased cytoplasmic FUS (Fig. 2C,D) following flavo treatment. These data provide evidence that anti-FUS antibodies recognizing an N-terminal epitope more consistently detect cytoplasmic FUS than those recognizing C-terminal epitopes, and are both consistent with and also provide an explanation for the previous discrepant results described above.

### FUS antibodies are specific to different isoforms

**Figure 3:**
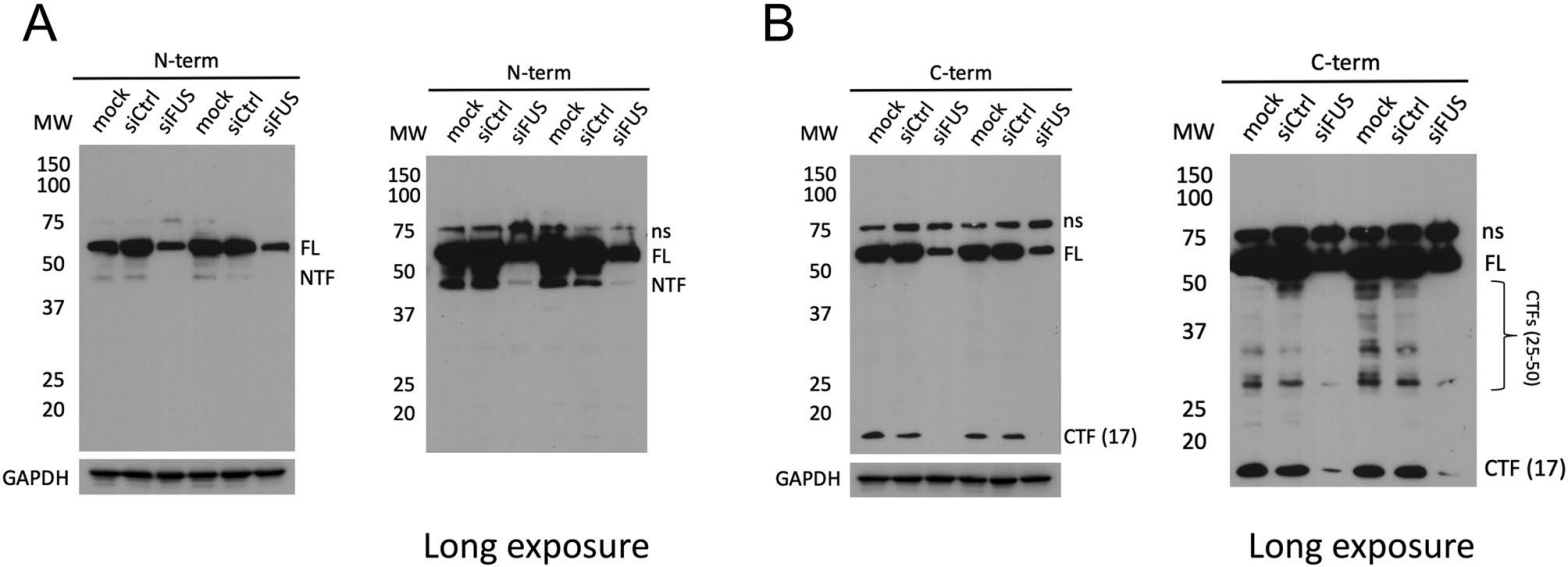
FUS isoforms are differentially recognized by N- and C-terminal antibodies. Western blot analysis of whole-cell lysates of U87 cells transfected with FUS siRNA. FUS immunoreactive bands were blotted with antibodies recognizing either an (**A**) N- terminal (1–50 aa) or (**B**) C-terminal (500–526 aa) epitope. FL: Full-length FUS; NTF: N-terminal fragment; CTF (17): C-terminal fragment at 17 kDa; CTFs (25–50): C-terminal fragments distributed from 25 to 50 kDa; ns: non-specific band. Uncut nitrocellulose membrane was first blotted with anti-FUS antibody and HRP-conjugated secondary antibody followed by film exposure. The same membrane was then washed with 0.1% TBST and incubated with anti-GAPDH antibody. GAPDH bands were acquired using Bio-Rad ChemiDoc MP imaging system. Original blots are shown in the Expanded file 2.

The above data revealed that the specificity of the antibody used to detect FUS in IF can affect the pattern observed. One explanation for these observations is that FUS produces truncated fragments and these may contribute to different IF signals. Indeed, human FUS was previously reported to be cleaved in transgenic flies^26^, and TDP-43 is well known to be subject to proteolytic cleavage^27,28^. To determine whether FUS fragments are present in human cells, we used WBs to analyze whole-cell lysates of U87 cells to determine if FUS immunoreactive species of lower molecular weights (full-length FUS migrates at ~ 68 kDa in 10% SDS-PAGE) could be detected. WBs using one of the antibodies recognizing an N-terminal epitope revealed a band around 45 kDa (Fig. 3A), while blots using an antibody directed against a C-terminal epitope displayed a major band at ~ 17 kDa as well as multiple minor bands between 25–50 kDa (Fig. 3B). All species were verified as FUS-related by FUS siRNA KD (Fig. 3A,B). We next expressed an N-terminal GFP-tagged FUS derivative (Tsai et al. 2020) in U87 cells (expression level ~ 1.9 times greater than endogenous FUS; see Supplemental Fig. S4A). WB analysis of GFP-FUS-transfected cells using an anti-GFP antibody also confirmed the presence of an N-terminal fragment (NTF) migrating near 75 kDa (Supplemental Fig. S4B). This is consistent with the size of the endogenous FUS NTF detected in Fig. 3A, which migrates around 45 kDa, fused with a 27 kDa GFP tag (Supplemental Fig. S4B). These findings reinforce the conclusion that FUS antibodies may not reliably detect FUS isoforms, at least in IF experiments.

### Transcriptional inhibition does not affect TNPO1-dependent FUS nuclear import

**Figure 4:**
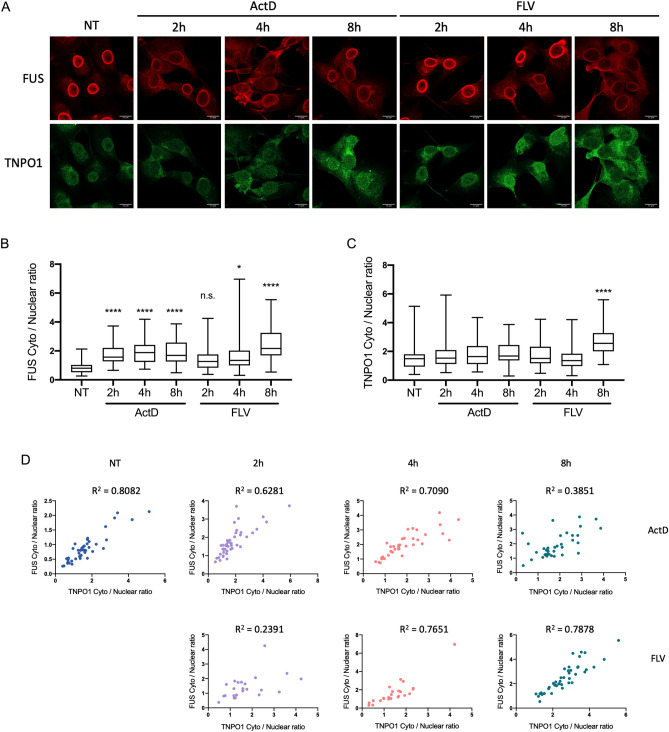
Nucleocytoplasmic localization of TNPO1 is largely unaffected by transcriptional inhibition. (**A**) U87 cells were untreated (NT) or treated with either 5 ug/mL ActD or 1 uM flavo for 2, 4 and 8 h followed by TNPO1 and FUS IF. Scale bars = 15 um. (**B**, **C**) Cytoplasmic to nuclear FUS (**B**) and TNPO1 (C) ratio quantification in untreated control, ActD- and FLV-treated cells. n = 23–45 cells were analyzed in each experimental condition. (*p = 0.0118, ****p < 0.0001) Dunn’s multiple test. (**D**) Pearson correlation analysis of FUS and TNPO1 cytoplasmic to nuclear ratios.

As mentioned in the Introduction, the nucleocytoplasmic distribution of the TNPO1 receptor shifts toward the cytoplasm and results in a FUS nuclear import defect upon hypertonic stress in neuronal cells^10^. To investigate whether TNPO1 localizes to the cytoplasm following transcriptional inhibition, we treated U87 cells with Act-D or flavo for 2, 4 or 8 h and analyzed the subcellular localization of FUS and TNPO1 by IF as above (Fig. 4A–C). In the Act-D-treated cells, FUS displayed striking cytoplasmic translocation from 2 to 8 h post-treatment, whereas TNPO1 did not show obvious changes in nucleocytoplasmic distribution (Fig. 4B,C). While flavo treatment for 8 h appeared to increase cytoplasmic TNPO1 levels, FUS cytoplasmic translocation occurred within 4 h, before the TNPO1 cytoplasmic shift (Fig. 4B,C). Therefore, these data suggest that increased FUS cytoplasmic translocation following transcriptional inhibition occurs independently of TNPO1 nucleocytoplasmic distribution.

While the above data indicates that aberrant FUS cytoplasmic localization is independent of TNPO1, we next investigated whether this is because TNPO1 is functionally defective in FUS nuclear import under the conditions studied. To address this, we performed a correlation test comparing TNPO1 and FUS cytoplasmic to nuclear ratios in individual U87 cells treated with or without transcriptional inhibitors for different times (Fig. 4D). In the non-treated cells, these ratios were strongly positively correlated (Pearson r = 0.8990, *p* < 0.0001), indicating normal TNPO1-dependent FUS nuclear import. Interestingly, the ratios were still significantly correlated in cells treated with either Act-D (Pearson r > 0.62; *p* < 0.0001) or flavo (Pearson r > 0.48; *p* < 0.0179) (Fig. 4D). These results suggest that TNPO1-dependent FUS nuclear import was largely unaffected by the inhibitors, and that FUS cytoplasmic accumulation reflects enhanced nuclear export, but not reduced import.

### Disruption of nuclear polyadenylated RNA export and decay pathways alters FUS nuclear-cytoplasmic distribution

**Figure 5:**
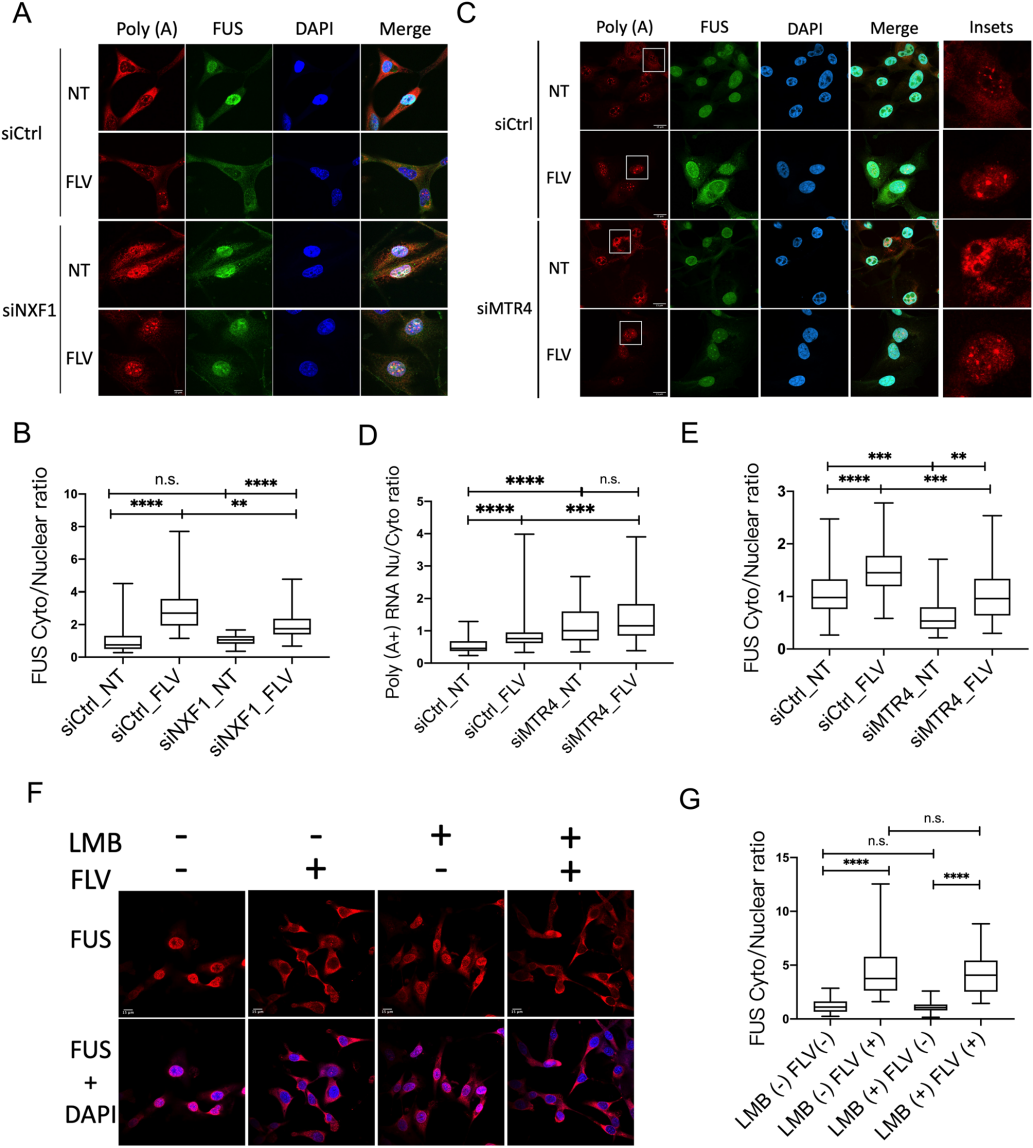
NXF1 and MTR4 knockdown, but not CRM1 inhibition, promotes FUS nuclear retention following transcriptional inhibition. (**A**) U87 cells were transfected with NXF1 siRNA for 48 h followed by poly(A +) RNA FISH and FUS IF. NT: No treatment; FLV: Flavo treatment (1 uM) for 4 h. Scale bar = 10 um. (**B**) Quantification of cytoplasmic to nuclear FUS ratios in siCtrl and siNXF1 transfected cells. n = 19–49 cells were analyzed in each experimental condition. (**p = 0.0033, ****p < 0.0001) Mann–Whitney *U*-test. (**C**) U87 cells were transfected with MTR4 siRNA for 72 h followed by poly(A +) RNA FISH and FUS IF. NT: No treatment; FLV: Flavo treatment (1 uM) for 4 h. Scale bar = 15 um. (D) Nuclear to cytoplasmic poly(A +) RNA ratios in siCtrl and siMTR4 transfected cells. n = 27–43 cells were analyzed in each experimental condition. (***p = 0.0007, ****p < 0.0001) Mann–Whitney *U*-test. (E) Quantification of cytoplasmic to nuclear FUS ratio in siCtrl and siMTR4 transfected cells. n = 27–37 cells were analyzed in each experimental condition. (**p = 0.0015, ***p = 0.0001–0.0004, ****p < 0.0001) Mann–Whitney *U*-test. (F) U87 cells were treated with flavo (1 µM) and/or leptomycin B (LMB, 40 nM) for 4 h followed by FUS IF. Scale bars = 15 um. (G) Cytoplasmic to nuclear FUS ratios of flavo- and/or LMB-treated cells. n > 20 cells were analyzed in each experimental condition. (****p < 0.0001) Mann–Whitney *U*-test.

We next examined if FUS cytoplasmic translocation in response to transcriptional inhibition is dependent on the nuclear mRNA export pathway. Although it was previously shown that FUS cytoplasmic localization following hypertonic stress is independent of mRNA export^10^, we wished to determine if this is also true after transcriptional inhibition. Notably, FUS interacts with SR protein splicing factors^29,30^, several of which can shuttle to the cytoplasm^31^ dependent on the mRNA export pathway and the nuclear RNA export factor NXF1^32,33^. We therefore depleted NXF1 using siRNA in U87 cells treated or not with flavo and quantified nucleocytoplasmic FUS levels by IF (Fig. 5A,B; knockdown (KD) efficiency shown in Supplemental Fig. S5A). Nuclear accumulation of poly(A +) RNA measured by oligo-dT FISH indicated successful abrogation of nuclear mRNA export (Fig. 5A). Notably, while the cytoplasmic to nuclear FUS ratio was unaffected after NXF1 KD in the untreated group, a significant increase in FUS nuclear retention was observed in the KD cells relative to controls following flavo treatment (Fig. 5A, quantitation in B). FUS nuclear export is considered to be a passive diffusion process^10,22^, and the fact that FUS nuclear retention was unaffected by NXF1 KD in untreated cells is consistent with this notion. However, because compromising the mRNA export pathway inhibits FUS cytoplasmic translocation following transcriptional inhibition, this pathway, either directly or indirectly, must participate in FUS nuclear export when RNAP II transcription is blocked.

As shown above, NXF1 KD leads to substantial nuclear poly(A +) RNA accumulation. Since FUS naturally binds RNA, we wondered if the global increase in potential binding substrates might be responsible for retaining FUS in the nucleus. The effect of disrupting the mRNA export pathway on FUS localization would then be indirect rather than reflecting a direct requirement for this pathway. To address this possibility, we KDed the nuclear exosome cofactor MTR4 (Supplemental Fig. S5B), which is an RNA helicase required for degradation of a subset of nuclear poly(A +) RNA^34,35^, and then examined both poly(A +) RNA levels and nucleocytoplasmic FUS distribution by IF with or without flavo treatment in control and KD cells. We indeed observed increases in nuclear to cytoplasmic poly(A +) RNA ratios after MTR4 KD, in both the untreated and flavo-treated cells (Fig. 5C, quantitation in Fig. 5D). Importantly, MTR4 KD cells showed reduced levels of cytoplasmic FUS (as judged by a reduced cytoplasmic/nuclear ratio) in both the untreated control cells and in cells treated with flavo (Fig. 5C, quantitation in Fig. 5E). We note that the increased nuclear to cytoplasmic poly(A +) RNA ratio (Fig. 5C,D) after flavo treatment was likely the result of cytoplasmic transcript degradation^36^, and not an actual increase in nuclear poly(A +) RNA concentration. Indeed, the nuclear poly(A +) RNA concentration measured by mean oligo-dT signal intensity (see “Materials and Methods”section) in the flavo-treated cells was decreased compared with untreated control cells (Supplemental Fig. S6). In any event, these results are consistent with those obtained with the NXF1 KD cells, and together our findings support the hypothesis that inhibition of RNAP II transcription results in elevated cytoplasmic FUS levels by decreasing the concentration of nuclear polyadenylated transcripts, which naturally serve to “anchor” FUS in the nucleus.

We next asked whether FUS transport to the cytoplasm following transcriptional inhibition requires the normal protein nuclear export machinery. Sequence predictions suggested that FUS contains a CRM1-mediated nuclear export signal (NES), but it was considered to be non-functional for FUS nuclear egress either under normal conditions or following hypertonic stress^10,22^. We tested if CRM1 is required for FUS export following transcriptional inhibition by treating U87 cells with both the CRM1 inhibitor leptomycin B (LMB) and flavo. We found that FUS underwent cytoplasmic translocation at levels undistinguishable between the LMB (+) and LMB (−) groups in the flavo-treated cells (Fig. 5F,G). As a control that the LMB was effective, we took advantage of the fact that the NES-containing substrate p53 is retained in the nucleus, and therefore activates target gene expression, when CRM1 is inhibited by LMB^37,38^. We observed increased dose-dependent p53 expression and elevation of the p53 target p21 in LMB-treated U87 cells (Supplemental Fig. S7A). Moreover, we found that LMB did not affect bulk nucleocytoplasmic poly(A+) RNA distribution within the time frame and LMB dosage used (Supplemental Fig. S7B). Therefore, our data shows that FUS cytoplasmic translocation following transcriptional inhibition occurs independently of CRM1-mediated nuclear export.

### Sporadic ALS patient cells display reduced expression of nascent transcripts

**Figure 6:**
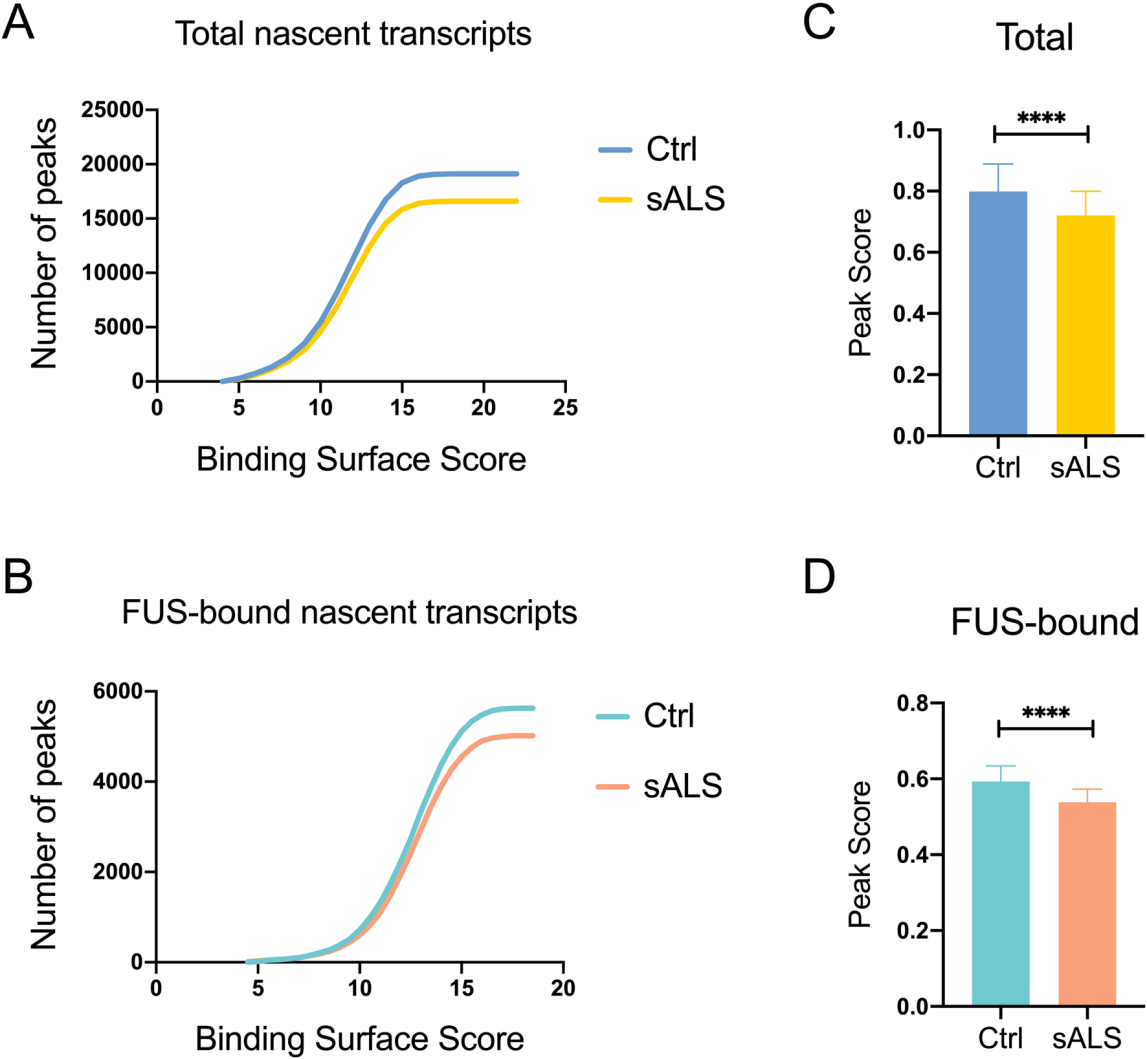
Nascent transcript levels are reduced in iPS cells derived from sporadic ALS patients Cumulative nascent transcript peak distribution of binding surface score of (A) total and (**B**) FUS-bound nascent transcripts in control (Ctrl) and sporadic ALS (sALS) iPSCs. (****p < 0.0001) Mann–Whitney *U*-test. (**C**, **D**) Mean expression level of (**C**) total and (**D**) FUS-bound nascent transcripts in control and sporadic ALS iPSCs. (****p < 0.0001) Mann–Whitney *U*-test.

Our data have shown that normal levels of RNAP II-dependent transcriptional activity are critical for maintaining nuclear localization of both FUS and TDP-43. Sporadic ALS patients commonly display TDP-43 cytoplasmic inclusions^8^, and it was recently found that FUS undergoes widespread mislocalization to the cytoplasm in such patients as well^5,39^. We thus wondered in light of the data described above whether RNAP II transcription might in some way be compromised in sporadic ALS patients. To address this question, we analyzed published nascent RNA-seq datasets obtained from two sporadic ALS patient iPS cell lines and two control cell lines reprogrammed from isolated fibroblasts^40^. Peak scores of all nascent transcripts were obtained by normalizing read counts to 10 million, and only read counts > 1 million were considered as true peaks. A total of 19,120 and 16,597 nascent transcript peaks were detected in the control and sporadic ALS patient iPSCs, respectively (Supplemental Table 1). We then used the combination of transcript lengths and expression levels to determine the potential “binding surface area,” which provides a measure of the cell’s ability to retain nuclear FUS by binding nascent transcripts. We next defined the logarithm of peak score multiplied by transcript length as the “binding surface score” (see “Materials and Methods”section for details of these calculations). Intriguingly, the sporadic ALS iPSCs not only showed reduced numbers of the detected nascent transcripts, but also presented less cumulative binding surface compared to the control iPSCs (Fig. 6A; Supplementary Table 1).

We next investigated whether known FUS-bound RNA targets in fact display reduced binding surface. To this end, we subset FUS-bound nascent transcripts based on published FUS CLIP-seq datasets of iPSC-differentiated motor neurons^41^ and performed binding surface score analysis. Indeed, the total binding surface score of FUS-bound nascent transcripts was reduced by ~ 10% in the sporadic ALS iPSCs compared to the control cells (*p* < 0.0001; Fig. 6B; Supplementary Table 2). Additionally, the averaged peak scores of total and FUS-bound nascent transcripts were also reduced, by ~ 10%, in the sporadic ALS samples (*p* < 0.0001; Fig. 6C,D; Supplementary Table 1 and 2). These findings, the possible significance of which is discussed further below, are consistent with our data that reduced levels of nuclear RNAP II transcripts lead to relocalization of FUS to the cytoplasm, and support our hypothesis that sporadic ALS patient cells are less able to retain FUS, and perhaps other nuclear RBPs, due to reduced production of nascent transcripts.

## Discussion

Cytoplasmic mislocalization and aggregation of TDP-43 and FUS are pathological hallmarks of ALS and FTD^4,5^. Indeed, TDP-43 pathology appears in > 90% of ALS and ~ 45% of FTD patients^8^. FUS mislocalization and aggregation, in contrast, is thought to occur mainly in familial ALS with *FUS* mutations and in ~ 10% of FTD^8^. However, recent findings have shown that FUS cytoplasmic mislocalization is a widespread feature of sporadic ALS^5,39^. Since *FUS* mutations are rare in ALS (< 0.1%) and their contributions to FTD remain elusive^42,43^, the mechanism responsible for wild-type FUS cytoplasmic translocation in ALS/FTD patients has been largely unclear. In this study, we report that nuclear mRNA metabolic processes, including transcription, export and degradation, can modulate FUS nucleocytoplasmic distribution in cultured cells. Importantly, and explaining how these findings have the potential to be relevant to ALS, we observed reduced nascent transcript levels in sporadic ALS iPS cell datasets compared to controls^40^, suggesting that widespread transcriptional disruption occurs in ALS and may contribute to FUS mislocalization pathology. Below we discuss these results and their significance in more detail.

FUS was previously suggested to exit the nucleus via passive diffusion. This conclusion was based on a protein enlargement assay, which measures cytoplasmic levels of FUS fused with different numbers of GFP tags. It was found that FUS with more GFP tags took longer to translocate to the cytoplasm, consistent with a diffusion model^10,22^. However, FUS is widely present in ribonucleoprotein complexes and interacts with numerous proteins/RNAs^21,41,44,45^. As these interacting partners possess different nucleocytoplasmic localizations, FUS distribution may well be affected by its binding partners. For example, FUS predominantly binds to pre-mRNA introns as demonstrated by CLIP-seq analysis^21,46^. Our data that transcriptional inhibition of RNAP II elicits FUS cytoplasmic translocation is consistent with the idea that RNA serves as a binding platform for FUS, thereby retaining the protein in the nucleus. This view was strengthened by our data showing increased FUS nuclear retention in NXF1 and MTR4 KD cells. While NXF1 KD leads to nuclear mRNA accumulation by inhibiting export, MTR4 KD mainly compromises RNA degradation. Additionally, while the majority of mature mRNAs are exported by NXF1^47^, MTR4 substrates in contrast include a range of pre-maturely terminated mRNAs, RNAP II-transcribed ncRNAs and other normally unstable nuclear RNAs^34,35^. Therefore, NXF1 and MTR4 KD are expected to induce accumulation of different subsets of nuclear RNA. Our data thus suggest that non-specific RNA accumulation in the nucleus is the main factor suppressing FUS cytoplasmic translocation following transcriptional inhibition, likely via increasing FUS binding targets. This proposal is consistent with the relatively promiscuous RNA binding properties of FUS^48^.

Our experiments revealed that the nature of the antibody used for FUS IF can affect measurements of nucleocytoplasmic localization. Although the basis for the differential staining patterns among the FUS antibodies we tested is not completely clear, it appears that antibodies recognizing C-terminal epitopes more effectively detect cytoplasmic FUS. As mentioned above, previous studies reported different results concerning FUS cytoplasmic translocation following transcriptional inhibition^10,19,20,22^, and our results offer a possible explanation for these findings. Additionally, our observations may at least partially explain other inconsistent results, regarding FUS nuclear export in response to stress^16,17^. We note that similar observations were made in human FUS transgenic flies stained with antibodies recognizing an N- or C-terminal epitope, with more cytoplasmic FUS observed with an antibody specific for a C-terminal epitope^26^. Since FUS cytoplasmic mislocalization is a pathological hallmark of disease, we suggest that use of mixed antibodies in IF may increase sensitivity and accuracy, and perhaps reveal novel FUS homeostatic pathways.

The N-terminal low complexity (LC) domain composes half of the FUS protein and plays a driving role in pathological aggregate formation^49^. Overexpression of full-length FUS or truncated FUS containing the LC domain induces cytotoxicity and aggregates in yeast and mouse models^50,51^. Therefore, FUS levels are tightly regulated via mechanisms such as endocytosis, proteasomal degradation and splicing-mediated autoregulation^52–54^. Our study also brings additional insights to FUS protein homeostasis. Our detection of N- and C-terminal FUS fragments indicates that the protein is a substrate for proteolytic cleavage. TDP-43 possesses similar biochemical properties as FUS and is cleaved by caspases in ALS and FTD patients^55–58^. We note that multiple consensus caspase cleavage sites can be found within FUS that are consistent with the NTF and CTF sizes we detected, suggesting that FUS can likely be cleaved by caspases as well. However, further investigation is required to confirm FUS as a caspase substrate and to determine whether its cleavage is important to ALS/FTD pathogenesis.

Our data has shown that reduced levels of nuclear RNA, which can reflect reduced transcription by RNAP II, result in increased accumulation of cytoplasmic FUS. The possible relevance of this to disease was suggested by the fact that iPS cells derived from ALS patients display reduced levels of transcription compared to normal controls. Indeed, based on these findings and previous results of others, we suggest a model by which an initial loss of FUS from the nucleus creates a “vicious cycle” that culminates with the substantial cytoplasmic accumulation that can result in FUS aggregates. The model is based on findings by Cech and colleagues that FUS binds to the RNAP II C-terminal domain and by doing so prevents premature phosphorylation of serine 2 (Ser2) residues by blocking access of the Ser 2-specific kinase CDK9/P-TEFb^59^. Loss of nuclear FUS thus allows Ser2 hyperphosphorylation, leading to RNAP II accumulation near transcriptional start sites. This was shown to occur on thousands of genes, resulting in premature polyadenylation and/or transcription termination^59^. We thus suggest that an initial event, such as a specific stress or transient decrease in transcription, leads to limited FUS cytoplasmic translocation. This in turn reduces FUS nuclear levels, perhaps at first incrementally, which allows increased Ser2 phosphorylation and impaired transcriptional elongation. The resulting decrease in nascent RNAP II transcripts and pre-mRNA favors FUS nuclear export and cytoplasmic accumulation by reducing FUS nuclear “anchors,” i.e., RNA. This results in further increases in Ser2 phosphorylation, and the cycle repeats. We note that the effect of reduced FUS levels on transcript levels reported by Schwartz et al.^59^ was modest, as was the reduction in nascent transcripts we detected in ALS iPSCs. This might however be consistent with a gradual accumulation of cytoplasmic FUS over an extended time period, conceivably contributing to the typically late onset of ALS.

We have proposed previously the concept of the “vicious cycle” (VC) in RBP pathology, and postulated how this can contribute to cellular dysfunction and ultimately cell death in ALS/FTD. We first observed in post-mortem brain samples from both *C9ORF72* (C9) repeat expansion-carrying patients (Conlon et al. 2016) and sporadic patients with no known mutation (Conlon et al. 2018) accumulation of insoluble RBPs^60,61^, which was accompanied by extensive missplicing. In the former case, aggregates appeared to be nucleated by binding of the splicing regulator hnRNP H to the transcribed C9 repeats, while in the later study we showed that multiple RBPs, including FUS, displayed insolubility. Analysis of the missplicing events revealed that transcripts encoding splicing-related RBPs were among the most dysregulated, suggesting how a VC might occur. An initial aggregation of an RBP could lead to limited missplicing, in turn leading to reduced levels of RBPs and/or RBPs more prone to aggregation (see Discussion in Conlon et al.^61^), more missplicing, and so forth. A separate analysis of missplicing in C9 brains described extensive intron retention (IR) that correlated with levels of insoluble hnRNP H^62^. Among the most enriched IR transcripts were those encoding components of the proteasome and autophagy pathways. This suggested another potential VC in which defects in protein quality control resulting from RBP aggregate-induced IR prevents clearance of aggregates, which allows increased buildup of such aggregates, more IR, further decreases in quality control, etc. Thus together with our results suggesting a possible FUS/transcription-based VC, we envision multiple ways in which initial RBP aggregates can amplify over possibly extended periods of time, functioning perhaps in concert or perhaps separately depending on the initial nucleating event, but in any case resulting ultimately in neuronal cell death and disease.

In summary, our study has provided considerable insight into factors that can cause FUS to translocate from nucleus to cytoplasm. Foremost among these are reductions in nuclear RNA concentration, for example due to reduced RNAP II transcription. Indeed, our analysis of sporadic ALS iPS cells has for the first time demonstrated a global reduction in nascent transcripts, suggesting a disruption of normal RNAP II transcriptional activity may occur in ALS patient brains. These findings offer an explanation for the increase in FUS cytoplasmic translocation recently found in sporadic ALS patients^5,39^, and provide a link between aberrant RNA synthesis and cytoplasmic localization of FUS in ALS/FTD. Future studies will examine the relevance of these findings to disease pathogenesis and progression.

## Methods

### Cell culture

U87-MG cells from American Type Culture Collection (ATCC) and primary human fibroblasts were cultured in DMEM (Gibco, cat # 11965092) supplemented with 10% FBS (Gemini, cat # 100-106) in a 37 °C, 5% CO_2_ incubator. Fibroblasts were a gift from Dr. Patricia Richard.

### Plasmid cloning and transfection

The GFP-tagged wild-type FUS plasmid was cloned using pEGFP-C3 vector as described in^63^. Lipofectamine 2000 (Thermo, cat # 11668019) and Dharmafect 1 (GE Dharmacon) were used for plasmid and siRNA transfection, respectively. Approximately 5 × 10^4^ U87 cells were seeded in each well of a 12-well plate. Transfection was performed 24 h post-seeding. Each well was transfected with 1 ug of plasmid DNA for 24 h or siRNA (Table 1.) with 15–20 nM final concentration for 48–72 h.

**Table 1:** siRNA sequences.

Target	Sense 5′→3′	Anti-sense 5′→3′	Supplier
FUS	CGGACAUGGCCUCAAACGA	UCGUUUGAGGCCAUGUCCG	GenePharma
NXF1	GCAAUUCAGGGCUAUGUAU	AUACAUAGCCCUGAAUUGC	Sigma
MTR4	CAAUUAAGGCUCUGAGUAA	UUACUCAGAGCCUUAAUUG	Sigma
Ctrl	UUCUCCGAACGUGUCACGU	ACGUGACACGUUCGGAGAA	GenePharma

### Pharmacological treatment

All pharmacological treatments were performed at least 24 h post cell seeding. Flavo (1 uM) or Act-D (5 ug/ml) final concentration were added to growth media for 4–6 h to inhibit transcription. DNA damage was induced by doxorubicin (0.5 uM) for 24 h. To induce oxidative stress, sodium arsenite (500 uM) was added to growth media for 1 h. Osmotic stress was induced by 0.4 M sorbitol treatment for 6 h. Leptomycin B (LMB, Alomone Labs cat # L-500) was diluted in growth media to final concentrations of 10 to 40 nM and incubated with U87-MG cells from 2 to 6 h depending on the specific experiment.

### Immunofluorescence and fluorescent in-situ hybridization (FISH)

Cells were fixed with 4% paraformaldehyde for 15 min followed by 0.3% Triton permeabilization for 20 min at 4 °C. Primary antibodies (Table 1.) were diluted in 3% BSA and incubated with fixed samples at 4 °C overnight. Fluorophore-conjugated secondary antibodies were then applied to the samples for 1 h at room temperature. Samples were washed with 0.2% PBS-T after each antibody incubation then mounted on microscope slides (Fluoroshield Mounting Medium with DAPI, cat # ab104139). Samples for poly (A+) RNA FISH were pre-incubated with 10% formamide at room temperature, then hybridized with biotinylated oligo(dT)_30_ (0.1 uM) in 10% dextran sulfate at 37 °C for 1 h, washed with 10% formamide and 2 × saline sodium citrate for 30 min, and incubated with fluorescent streptavidin at room temperature followed by 0.2% PBS-T washes and mounted on microscope slides.

### Nucleocytoplasmic fractionation

U87 cells were washed with 1xPBS and pelleted. Five times volume of cytoplasmic extraction (CE) buffer was added to the cell pellet and rotated at 4 °C for 5 min to lyse the cells. Lysates were centrifuged at 3000 rpm at 4 °C for 5 min and the supernatant collected (CE fraction). Nuclear pellets were washed with CE buffer without NP-40 and resuspended in an equal volume of nuclear extraction (NE) buffer then incubated on ice for 10 min, with occasional vortexing. Nuclear extracts (NE) were centrifuged at 4 °C at 14,000 rpm for 5 min, the supernatant collected (NE fraction) and the pellet resuspended in NE buffer (chromatin fraction). Samples were then mixed with 4 × SDS protein sample buffer and boiled for 10 min for subsequent WB analysis. CE buffer composition: HEPES 10 mM pH 7.9, KCl 10 mM, EDTA 0.1 mM, NP-40 0.3%, 1 × protease inhibitor cocktail. NE buffer composition: HEPES 20 mM pH7.9, NaCl 400 mM, EDTA 1 mM, Glycerol 25%, 1 × protease inhibitor cocktail.

### Western blotting

Cells were lysed with 1 × RIPA buffer (150 mM NaCl, 50 mM Tris pH 8.0, EDTA 1 mM, 0.25% Sodium deoxycholate, 1% NP-40) supplemented with 1 × protease inhibitor cocktail on ice for 5 min and boiled after addition of appropriate amount of 4 × SDS protein sample buffer for 10 min. Protein samples were resolved by SDS-PAGE and then transferred to a nitrocellulose membrane. Membranes were blocked with Pierce protein-free blocking buffer (Thermo, cat # 37,570) followed by incubation with primary antibodies (Table 2) at 4 °C overnight and washed with 0.1% TBS-T at least three times. All primary antibodies were diluted in the blocking buffer based on instructions of antibody manufacturers. Secondary antibodies were applied to the membrane the next day at room temperature for 1 h and washed with 0.1% TBS-T. Protein bands were developed using ECL HRP substrate (Millipore) then imaged with X-ray films or Bio-Rad ChemiDoc MP imaging system. Original blots for all figures are provided in the Supplementary information file. Edges are shown in all blots exposed with X-ray films. Images in Expanded File 2, 6 and GAPDH bands of Expanded File 4 were imaged with Bio-Rad ChemiDoc MP imaging system, which does not produce edges. Primary and secondary antibody incubations were carried out on uncut nitrocellulose membranes in Fig. 3, Supplemental Fig. S4 and MTR4 of Supplemental Fig. S5B. For Supplemental Fig. S1, S5A and S7A, nitrocellulose membranes were cut within the range of target protein molecular weights prior to primary and secondary antibody incubation.

**Table 2:** Antibodies.

Antibody	Supplier	Cat #	Epitope
FUS	Santa Cruz	sc-373698	2-27 aa
	Bethyl	A300-302A-T	1-50 aa
	Santa Cruz	sc-47711	C-term
	Bethyl	A300-294A-T	500–526 aa
TDP-43	Proteintech	10782-2-AP	
hnRNP C	Proteintech	11760-1-AP	
TNPO1	Proteintech	20679-1-AP	
NXF1	Santa Cruz	sc-32319	
GAPDH	Sigma	G9545-200UL	
GFP	ABM	G095	
MTR4	Novus	NB100-1575	
p21	CST	2947	
p53 (1801 DO1)	Gift from Dr Carol Prives Lab		
G3BP1	Proteintech	13057-2-AP	
ACTB	Sigma	AV40173	
Histone 3	Abcam	ab12079	

### Image acquisition and analysis

Immunofluorescence and FISH samples were imaged with a Zeiss LSM700/800 confocal microscope and analyzed with Fiji ImageJ. To measure cellular FUS protein levels after siRNA transfection, fluorescent images were acquired using the same laser power and gain across the transfected samples. Total FUS fluorescent intensity per cell was obtained using integrated density (mean pixel intensity × cell area or region of interest (ROI)). We note that uneven cell focal planes in a transfected sample could lead to biased estimation of cellular FUS level. Therefore, total FUS intensity per cell was further normalized to DAPI intensity to obtain the adjusted cellular FUS protein level. To determine cytoplasmic-nuclear protein ratios, whole-cell and nuclear integrated densities of the indicated protein were first obtained. The nuclear integrated density was then subtracted from the whole-cell integrated density to give the cytoplasmic protein level. We then divided cytoplasmic by nuclear integrated densities to acquire cytoplasmic to nuclear protein ratios. To obtain background-adjusted fluorescent intensities of a frame, we first measured the mean fluorescent intensity of a staining-free ROI. Next, we subtracted the integrated density of antibodies or FISH staining from the mean background intensity multiplied by cell area, which results in fluorescent intensity values with less background interference. To obtain relative nuclear poly(A+) RNA concentrations in untreated control and flavo-treated cells, total nuclear poly(A+) RNA intensity are adjusted to DAPI and divided by the area of the nucleus.

### Nascent RNA-seq re-analysis

Bru-Seq datasets of control and sporadic ALS (sALS) iPSCs were downloaded from GSE115310^40^. Raw reads were mapped to human genome hg19 using bowtie 2. Nascent transcripts were identified using the HOMER GRO-Seq program makeTagDirectory command to generate 191404803 sequence tags for the control samples and 166632004 tags for sALS samples^64^. Next, we ran findPeaks command using the generated tags and identified 47148 nascent transcript peaks in control and 41232 peaks in sALS patients. To select true nascent transcript peaks, reads of each peak region was further normalized to 10 million total aligned reads to obtain peak scores, and peak scores smaller than 0.1 (< 0.0001% total aligned reads) were discarded, which left 19120 and 16597 transcript peaks in control and sALS, respectively. To assess the cell’s ability to retain FUS in the nucleus by association with pre-mRNAs, we took advantage of the fact that FUS binds RNA in a length-dependent manner^48^, so the length of a nascent transcript is positively correlated with the amount of FUS it could possibly tether. As Bru-Seq obtains only RNA incorporating bromouridine, we assume the length of an identified peak region would be the length of an actively transcribed transcript. We further multiplied the length of a nascent transcript by its expression level to reflect the total FUS protein it could tether. We define this measure as “Binding surface score = log_2_(peak score × transcript length)”. To obtain nascent transcripts that are FUS binding targets, we subset gene names from Supplementary Table 1 that overlap with wild-type FUS binding targets in iPSC-derived motor neurons reported by De Santis et al.^41^. The results of the re-analysis were provided in Supplementary Tables 1 and 2.

## Supplementary Information

Supplementary Table 1.

Supplementary Table 2.

Supplementary Figures.

## Data Availability

The datasets analyzed during the current study are available in Gene Expression Omnibus (GEO; Accession number: GSE115310). The results of the analysis are included in this published article and its Supplementary Information files. No datasets were generated during the current study.
