# Supplementary material for: Nuclear RNA transcript levels modulate nucleocytoplasmic distribution of ALS/FTD-associated protein FUS
Source: Sci Rep. 2022 May 17;12:8180. doi: 10.1038/s41598-022-12098-4 (PMC9114323; doi:10.1038/s41598-022-12098-4)
Supplement: Supplementary file 3 — Supplementary Figures. [file 41598_2022_12098_MOESM3_ESM.docx]

**Supplemental Figures**

**Supplemental Figure S1. Nucleocytoplasmic fractionation of U87 cells.** U87 cells were incubated with 1 uM flavopiridol for 16 hours followed by nucleocytoplasmic fractionation. ACTB and H3 were used as cytoplasmic and nuclear markers, respectively. NT: Untreated; FLV: Flavopiridol-treated; CE: Cytoplasmic extract; NE: Nuclear extract; Chr: Chromatin fraction. ACTB: Beta-actin; H3: Histone 3. Uncropped blot is in Expanded file 1.

**Supplemental Figure S2. DNA damage, oxidative stress and serum deprivation do not induce FUS cytoplasmic translocation**. (A) U87 cells were incubated with 0.5 uM doxorubicin for 24 hrs followed by FUS IF. Cytoplasmic to nuclear FUS ratios were quantified. n>20 cells were analyzed in each experimental condition. Mann-Whitney *U*-test revealed no significant difference (ns). (B) U87 cells were incubated with 500 uM sodium arsenite for 1 hr followed by FUS and (C) G3BP1 IF and cytoplasmic to nuclear FUS ratio analysis. n>25 cells were analyzed in each experimental condition. (****p <0.0001) Mann-Whitney *U*-test. Inset arrows: FUS granules. Inset white lines: Cell boundary. (D) Human fibroblasts were incubated with 500 uM sodium arsenite for 1 hr followed by FUS and G3BP1 IF staining. (E) U87 cells were incubated in normal or serum-deprived media for 6 hrs followed by FUS IF and cytoplasmic to nuclear FUS ratio analysis. n=11~13 cells were analyzed in each experimental condition. Columns are shown in means with standard deviations. Mann-Whitney *U*-test. Scale bars = 15 um.

**Supplemental Figure S3. Antibodies used for immunofluorescence are specific to FUS epitopes.** (A) Secondary antibody staining background correction. FUS was stained with one antibody but incubated with rabbit (green) and mouse (red) host secondary antibodies mixture. The obtained background signals were subsequently used for background subtraction. (B) U87 cells were mock transfected or transfected with siCtrl or siFUS as indicated for 72 hrs followed by FUS IF using a mixture of an anti-FUS antibody recognizing the N-terminus (a) and an anti-FUS antibody recognizing the C-terminus (d). (C) Total FUS fluorescence intensity quantification normalized to DAPI. n=21~23 cells were analyzed in each experimental condition. (**p =0.0081, ***p =0.0002, ****p <0.0001) Mann-Whitney *U*-test. (D) U87 cells were transfected with siFUS for 72 hrs followed by FUS IF using a mixture of an anti-FUS antibody recognizing the N-terminus (b) and anti-FUS antibody recognizing the C-terminus (c). (E) Total FUS fluorescence intensity quantification normalized to DAPI. n=22~26 cells were analyzed in each experimental condition. (****p <0.0001) Mann-Whitney *U*-test. Scale bars = 15 um.

**Supplemental Figure S4. Exogenous expression of GFP-FUS produces N-terminal fragment (NTF).** Plasmids encoding GFP alone (GFP) or a GFP-FUS fusion (WT) were transfected into U87 cells for 36 hrs followed by western blot analysis using (A) anti-FUS or (B) anti-GFP antibodies. GFP-NTF at ~ 75 kDa was detected below the full-length GFP-FUS (~100 kDa). Endo FUS: Endogenous FUS. In (B), uncut nitrocellulose membrane was first blotted with anti-GFP antibody and HRP-conjugated secondary antibody followed by film exposure. The same membrane was then washed with 0.1% TBST and incubated with anti-GAPDH antibody. GAPDH bands were acquired using Bio-Rad ChemiDoc MP imaging system. Full blot for (A) in Expanded file 3; Full blot for (B) in Expanded file 4.

**Supplemental Figure S5. Evaluation of NXF1 and MTR4 knockdown using siRNA.** U87 cells were transfected with siNXF1 or siMTR4 for 48-72 hrs followed with flavo (1uM, 4 hrs) treatment. The western blot shows the extent of (A) NXF1 and (B) MTR4 knockdown. Relative expression of NXF1 or MTR4 in each lane was normalized to GAPDH band intensity. In (B), uncut nitrocellulose membrane was first blotted with anti-MTR4 antibody and HRP-conjugated secondary antibody followed by Bio-Rad ChemiDoc MP imaging system acquisition. The same membrane was then cut and washed with 0.1% TBST and incubated with anti-GAPDH antibody. GAPDH bands were acquired using Bio-Rad ChemiDoc MP imaging system. Full blot for (A) in Expanded file 5; Full blot for (B) in Expanded file 6.

**Supplemental Figure S6. Quantification of mean nuclear poly(A+) RNA signal intensity.** The quantification is associated with Fig. 5. U87 cells were treated with or without flavo (1 uM, 4 hrs) followed by poly (A+) RNA FISH. Images were acquired using the same laser power and gain. Poly (A+) RNA fluorescent intensities of untreated (NT) and flavo (FLV) treated cells were adjusted to DAPI and nuclear area to obtain mean signal intensities. n=22~26 cells were analyzed in each experimental condition. (**p=0.0014) Mann-Whitney *U*-test.

**Supplemental Figure S7. Evaluation of leptomycin B (LMB) efficacy and its effects on poly(A+) RNA distribution.** (A) Western blot analysis of U87 cells verifying p53 stabilization and p21 activation in response to LMB (0, 10, 20 and 40 nM). Full blot in expanded file 7. (B) Poly (A+) RNA FISH of U87 cells incubated with LMB 40 nM for 0, 2, 4 and 6 hrs.

**Supplementary Table 1**: Binding Surface Scores (BSS) from Bru-Seq datasets of control and sporadic ALS iPS cells.

**Supplementary Table 2**: Binding Surface Scores (BSS) of FUS-bound transcripts from Bru-Seq datasets of control and sporadic ALS iPS cells.

**Expanded Files**

Expanded File 1

Expanded File 2

Expanded File 3

Expanded File 4

Expanded File 5

Expanded File 6

Expanded File 7
